# Intake of high fructose corn syrup sweetened soft drinks, fruit drinks and apple juice is associated with prevalent coronary heart disease, in U.S. adults, ages 45–59 y

**DOI:** 10.1186/s40795-017-0168-9

**Published:** 2017-06-27

**Authors:** Luanne Robalo DeChristopher, Jaime Uribarri, Katherine L. Tucker

**Affiliations:** 1 Biochemistry, Molecular Biology, P.O. Box 5542, Eugene, OR 97405 USA; 2 Department of Medicine, the Icahn School of Medicine at Mount Sinai, New York, NY USA; 3 Department of Biomedical and Nutritional Sciences, University of Massachusetts Lowell, Lowell, MA USA

**Keywords:** Excess free fructose, Fructose, Coronary heart disease, Heart disease, Fructose malabsorption, Advanced glycation end products, Fructositis, High fructose corn syrup, HFCS, enFruAGE, AGE, RAGE, Sugar sweetened beverages, SSB, Isoglucose, Fructose glucose ratio

## Abstract

**Background:**

Intake of high excess free fructose (EFF) beverages, including high fructose corn syrup (HFCS), sweetened soft drinks, fruit drinks, and apple juice, may be associated with childhood asthma, adult idiopathic chronic bronchitis/ COPD, and autoimmune arthritis, possibly due to underlying fructose malabsorption. Fructose malabsorption may contribute to the intestinal in situ formation of advanced glycation end-products (enFruAGEs) that travel to other tissues and promote inflammation. Chronic respiratory conditions and arthritis are comorbidities of coronary heart disease (CHD). The objective of this study was to investigate the association between intake of high EFF beverages and CHD.

**Methods:**

In this cross sectional study (NHANES 2003–2006) of adults, aged 45–59 y, *n* = 1230, the exposure variables were non-diet soft drinks, and any combination of high EFF beverages including non-diet soft drinks, fruit drinks, and apple juice. Analyses of diet soft drinks, diet fruit drinks, and orange juice (non/low EFF beverages) were included for comparison. The outcome was self-reported history of coronary heart disease and/or angina (CHD). Rao Scott Ҳ^2^ was used for prevalence differences and logistic regression for associations, adjusted for age, sex, race-ethnicity, BMI, socio-economic status, health insurance coverage, smoking, physical activity level, hypertension, energy intake, fruit and vegetable intake, glycated hemoglobin, pre-diabetes, and diabetes.

**Results:**

Intake of any combination of HFCS sweetened soft drinks, fruit drinks, and apple juice (tEFF) was significantly associated with CHD in adults aged 45–59 y. Adults consuming tEFF ≥5 times/wk. were 2.8 times more likely to report CHD than ≤3 times/mo consumers (OR 2.82; 95% CI 1.16–6.84; *P* = 0.023), independent of all covariates.

**Conclusion:**

HFCS sweetened soft drinks, fruit drinks, and apple juice may contribute to CHD, a common comorbidity of chronic respiratory conditions and autoimmune arthritis, possibly due to the high ratio of fructose to glucose in these beverages. Underlying fructose malabsorption may contribute to the intestinal in situ formation of pro-inflammatory enFruAGEs, that are eventually absorbed and induce inflammation of the coronary arteries. Additional research is needed.

**Electronic supplementary material:**

The online version of this article (doi:10.1186/s40795-017-0168-9) contains supplementary material, which is available to authorized users.

## Background

The etiology of many chronic, comorbid, pro-inflammatory diseases remains unknown [1–5]. Research that aims to explain why chronic respiratory conditions, including chronic obstructive pulmonary disease (COPD) [6, 7] and asthma [8], are common comorbidities of cardio-metabolic diseases and autoimmune arthritis is an active area of ongoing investigation [6–9]. According to the US Centers for Disease Control and Prevention, chronic respiratory conditions, including idiopathic chronic bronchitis – a subcomponent of COPD - are common comorbidities of type 2 diabetes (T2D), heart disease, and arthritis [10, 11]. Recent research by the Mayo Clinic indicates that children with asthma have increased risk of T2D, and adults with asthma have increased risk of T2D and coronary heart disease (CHD) [9].

How intake of sugar sweetened beverages (SSB) is contributing to epidemic levels of these diseases is also an active area of ongoing research [12–17]. Individuals who regularly consume SSB have increased risk of CHD [15, 16], seropositive rheumatoid arthritis (RA) [17], and T2D [12–14]. One proposed hypothesis for these associations is that the high dietary glycemic load from SSB leads to chronic inflammation, insulin resistance, and impaired ß-cell function [12–17]. However, this hypothesis is unsupported in epidemiologic research, as large-scale longitudinal studies have shown that adults who regularly consumed SSB had increased risk of CHD [15, 16] and RA [17] that was independent of risk factors known to be associated with CHD and RA, including weight gain, high blood pressure, smoking, and of T2D [15–17].

Although most prospective studies with SSB and T2D did not adjust for glycemic load [12], results from two large-scale studies showed that the increased risk of T2D among regular consumers of SSB persisted, even after adjustments for lifestyle and dietary factors that included glycemic load [18, 19]. This raises the possibility that another mechanism – other than glycemic load and ß-cell fatigue – may be contributing to the increased risk of T2D and its comorbidities, among regular consumers of SSB.

Another hypothesis which aims to explain the association between regular intakes of SSB and increased risk of these comorbid diseases, is that the advanced glycation end-products (AGE) present in the caramel coloring of soda, contribute to chronic inflammation [12–17]. Although there is consistent evidence that AGE contribute to atherosclerotic plaque, and increased risk of heart disease [20], this hypothesis is unsupported, as diet soda contains the same caramel coloring as non-diet soda and, in largescale longitudinal studies, diet soda was *not* found to be associated with CHD [15, 16], or with RA [17]. Results with T2D have been mixed, possibly due to residual confounding [12, 21]. It is also noteworthy that, relative to other foods, soft drinks *have not* been found to be a significant source of pro-inflammatory dietary AGE [22].

One overlooked source of AGE, independent of glycemic load and blood glucose concentration, which could explain the increased risk of T2D, CHD and RA among regular consumers of SSB, is intestinally formed AGE [23]. It has been hypothesized that the association between SSB and prevalence of these chronic comorbid diseases may be due to underlying fructose malabsorption, which results after consumption of foods with a high fructose to glucose ratio (unpaired excess free fructose) – as occurs in high fructose corn syrup - but not after consumption of sucrose. Consumption of high fructose to glucose ratios results in elevated amounts of unabsorbed fructose in the gut that may contribute to the intestinal in situ formation of AGE [23–27]. Importantly, emerging science provides evidence that there may be a metabolic connection of unknown etiology with each of these comorbid conditions (asthma [28], chronic bronchitis [24], autoimmune arthritis [25], T2D [29], and CHD [30]), and that each should be redefined as a multi-system disorder involving the gut.

Over the past 30 years, HFCS has been the main sweetener in US soft drinks and fruit drinks [31], and there is evidence that the amount of fructose in the HFCS used to sweeten SSB may be higher than is generally recognized as safe (GRAS) [32–34]. Two separate studies showed that fructose comprised 65% [32] and 60% [33] of the sugars in soft drinks - not the 55% that is GRAS [34]. The objective of this cross sectional study was to investigate the association between intake of HFCS sweetened beverages (SSB), apple juice and CHD. Apple juice is included in the analysis, as its fructose to glucose ratio is approximately 2:1, versus about 1:1 in orange juice [35]. Analyses with non/low EFF beverages (orange juice, diet drinks) were included for comparison. A large nationally representative survey dataset – the National Health and Nutrition Examination Survey (NHANES) – was utilized for years 2003–2006 [36]. During this data collection period, HFCS was the predominant sweetener in US soft drinks [31].

## Methods

### Design overview

The NHANES assesses the health and nutritional status of adults and children in the United States. Data were used to analyze the correlation between intake of beverages with high fructose to glucose ratios, (non-diet soda, apple juice and fruit drinks) and prevalent CHD. We included fruit drinks in our analyses, as fruit drinks often contain naturally high EFF apple juice or are sweetened with HFCS. The NHANES combines interviews and physical examinations and, from 2003 to 2006, included a food frequency questionnaire (FFQ). Strong and consistent relationships have been reported between frequency of food and food-group consumption and probability of consumption on 24-h recalls [37]. FFQ have therefore been used as reliable sources of food intake and dietary patterns in epidemiological research [38–40].

The NHANES uses a complex sampling design and constructs sample weights to produce nationally representative data [41]. The sample weights provided in the food frequency data files were used for statistical analyses. They account for oversampling of various groups and reflect the fact that, of the 20,470 people who participated during the 4 year period, not all participated in the FFQ. Therefore, statistics and summary tables are appropriately weighted to account for oversampling. All NHANES participants provided informed consent, and the NHANES protocol was approved by the Research Ethics Review Board of the Centers for Disease Control and Prevention’s (CDC) National Center for Health Statistics (NCHS). More detailed information on NHANES is available online [34]. For this cross sectional study, there was no patient involvement.

Overall, 11,505 individuals, ages 2 to 85 y, responded to the FFQ beverage intake questions and participated in the medical examination component (MEC) of the survey, and 6240 provided information regarding CHD history. The focus of this analysis was adults aged 45–59 y, as CHD is most prevalent among older adults [42] and, among older adults, those under 60 are most likely to consume soft drinks and fruit drinks [43]. We limited our analyses to adults ages 45–59 y because, not only does soft drink consumption decline significantly among adults 60 and older, patients aged ≥60 y will have had significantly less HFCS exposure than younger study participants. This is because circa 1984, major soft drink producers switched from sugar to HFCS to sweeten US soft drinks [44].

In 1984, study participants aged ≥60 y were ≥40 y. Importantly, adults ages 45–59 y have the second lowest SSB intake (40–59 y old: men, 159 kcal p/d; women, 86 kcal p/d) [43] relative to younger adults (20–39 y old: men, 252 kcal p/d; women, 138 kcal p/d) [43] and adolescents (12–19 y old: boys, 273 kcal p/d; girls, 138 kcal p/d) [43]. Therefore, individuals 60 y and older in 2003 (beginning of the study period) would have consumed HFCS for a shorter duration than younger adults. Notably, they are also the lowest consumers of SSB (60 y ≥: men, 70 kcal p/d; women 42 kcal p/d) [43] relative to all other age groups.

In association studies of CHD, exposure duration is an important consideration, as diets that contribute to plaque formation do so over time - beginning as early as childhood [45]. Annual average per capita HFCS consumption was approximately 30 dry pounds in 1984. It steadily increased to more than a pound per week in 1999, and has since declined to just under a pound per week [46, 47]. Of the 2023 adults aged 45 to 59 years who provided information regarding CHD, 1209 adults provided complete responses to all demographic, medical examination and high EFF beverage intake questions.

### Variables

The outcome variable was self-reported CHD or history of CHD, or angina (pectoris) or history of angina [33]. The exposure variables were consumption frequency of any combination of HFCS sweetened soft drinks, fruit drinks, and apple juice, herein referred to as tEFF; or consumption frequency of non-diet soft drinks; diet soft drinks and diet fruit drinks; and orange juice. High energy drinks and sweetened teas – drinks that are often included in epidemiologic research of SSB – were not included in our analyses, as many varieties are sweetened with sucrose and/or glucose. Therefore, they contain a ≤ 1:1 ratio of fructose to glucose, and are *not* high EFF beverages [48]. In crude analyses, 1359 participants responded to questions regarding tEFF, non-diet soft drinks and orange juice, and 1273 and 969 participants responded to questions regarding diet soft drinks and diet fruit drinks, respectively. In the NHANES FFQ, consumption frequency of orange juice was combined with grapefruit juice [36]. Grapefruit juice, like orange juice, contains approximately a 1:1 ratio of fructose and glucose and is, therefore, a low EFF juice [35].

The FFQ questions regarding soft drink consumption distinguished between (non-diet, diet, caffeine free and caffeinated) soda consumed in the summer versus the rest of the year [36]. These were summed, and the seasons averaged to calculate average daily intake frequencies for non-diet and diet soft drinks. The CDC assigned frequencies for beverages using the following (NCI Dietcalc software) algorithm: Never = 0; 1 time per month or less = 0.03; 2–3 times per month = 0.08; 1–2 times per week = 0.21; 3–4 times per week = 0.5; 5–6 times per week = 0.79; 1 time per day = 1; 2–3 times per day = 2.5 [36]. For analyses, we combined intake frequencies of all beverages into categories as follows: ≤ 3 times/mo (reference group); 1–4 times/wk.; and ≥5 or more times/wk. To minimize missing data, intake frequency was obtained from raw data and Dietcalc files, as Dietcalc files contained missing data if an individual answered no to the lead question, “Did you drink soft drinks?”, and then provided answers to intake questions. For analyses of any combination of high EFF beverages, (apple juice, fruit drinks, and non-diet soda), we assigned each beverage zero for once a month or less; 0.117 for 2–3 times a month; 0.357 for 1–4 times per week; and 1 for ≥5 times per week. We then summed these values to establish intake frequencies as described above

Adjustment variables included sex, race/ethnicity, age, body mass index, energy intake, smoking, family income to poverty ratio, education level, health insurance coverage, physical activity, blood pressure, fruit and vegetable intake - a measure often used as a barometer of healthy lifestyle, pre-diabetes, diabetes, and glycated hemoglobin (A1c) - a measure of the average blood glucose over the previous 3 months. Adjustment variables were selected for use in this study based on existing research [15, 16]. However, unlike most epidemiologic analyses of SSB and CHD [15, 16], glycated hemoglobin and pre-diabetes were included to assess the association between intake of high EFF beverages and CHD, independent of blood glucose concentration.

Total energy intake and total fruit and vegetable intake were obtained from the 24-h dietary recall and calculated using either the average of two 24-h recalls or one 24-h recall if the second recall was missing. Fruit and vegetable intake (g/day) included all USDA (fruit) food codes for citrus fruits (611×), dried fruits (62×), other fruits (63×), and excluded citrus fruit juices, fruit juices/nectars excluding citrus; and included all USDA (vegetable) food codes for white potatoes/potato salad/potato recipes/potato soups/Puerto-Rican starchy vegetables (71×), dark green vegetables (72×), deep-yellow vegetables (73×), tomatoes and tomato mixtures, except tomato juice (74×), other vegetables (75×, 76×), and vegetables with meat, poultry and fish (77×).

Body mass index (BMI, Kg/m^2^) was calculated by the NHANES, from measured height and weight. Pre-diabetes was defined as A1c ≥ 5.7% and ≤6.4%. Diabetes was self-reported (Doctor said you have diabetes). Smoking and history of smoking were self-reported with a series of questions including, “Do you now smoke cigarettes? During the past 5 days, did you use cigarettes?” Have you smoked at least 100 cigarettes in your entire life? [36] For analysis purposes these were used to distinguish between non-smokers, non-smokers with a history of smoking, and current smokers. Adjustment for socioeconomic status (SES) included data obtained for family income to poverty ratio and respondent education level [36]. For analysis purposes, categories used for family income to poverty ratio were below 1, 1–1.99, 2–3.99, and 4 and above; and education levels were reduced to HS/GED and below, and some college and above.

The NHANES obtained information on physical activity and provided recommended metabolic equivalent (MET) scores for each activity [36]. For analysis, MET scores were summed and divided into tertiles. The physical activity questions and corresponding MET scores are described in Additional file 1: Table S1. The NHANES provided the average of three consecutive blood pressure readings for systolic and diastolic blood pressure. Respondents who smoked, consumed coffee, or alcohol within 30 min prior to having their blood pressure measured (48) were excluded from analyses. Respondents were classified as having hypertension if their systolic blood pressure was equal to or above 140, their diastolic blood pressure was equal to or above 90, or if they were taking prescription medication for hypertension [36].

### Statistical analysis

Analysis was performed with revision 13 of statistical software from the Stata Corporation. Rao Scott Ҳ^2^ analysis was used to test for significance of differences in CHD prevalence by beverage intake frequency. A *p*-value <0.05 was considered significant. Logistic regression was used to assess the adjusted odds ratio between exposure variables and CHD, independent of potential confounding variables.

In addition to a crude model, two multivariable regression models were used to assess the association between intakes of any combination of HFCS sweetened soft drinks, fruit drinks, and apple juice (tEFF) and CHD prevalence. The first multivariable model adjusted for age, sex, race/ethnicity, BMI, SES, health insurance coverage, energy intake, fruit and vegetable intake, physical activity level, smoking, history of smoking, and hypertension. The second model also adjusted for glycated hemoglobin (A1c), pre-diabetes, and diabetes. For analyses of non-diet soft drinks, further adjustment was made for apple juice and fruit drinks, to assess the association between HFCS sweetened soft drinks independent of these other high EFF beverages. In logistic regression analysis, confidence intervals that did not include 1 and *p* values <0.05 were considered statistically significant.

## Results

Overall, 5.0% of 1230 adults aged 45–59 y reported that they had CHD (Table 1). In this cross sectional study, the prevalence of CHD among adults, aged 45–59 y, consuming any combination of HFCS sweetened soft drinks, fruit drinks and apple juice ≥5 times/wk. (7.9%) was more than double that of ≤3 times/mo consumers (3.3%) (*P* = 0.044). Similarly, the prevalence of CHD among ≥5 times/wk. consumers of HFCS sweetened soft drinks was more than double (8.7%) that of ≤3 times/mo consumers (4.1%) (*P* = 0.044) (Table 2). No associations were seen between regular intake of diet soft drinks, diet fruit drinks, or citrus juice and prevalence of CHD (Tables 2, 3 and 4). In fact, CHD prevalence among regular consumers of citrus juice (≥5 times/wk) was markedly lower (5.0%) than that of regular consumers of tEFF (7.9%) or of non-diet soft drinks (8.7%) (Table 2).

**Table 1:** Characteristics of Adults, aged 45–59 years in the NHANES 2003–2006

n	1230
Age (y, mean ± SD)	51.7 ± 3.2
Sex (% male)	47.6
Race/ethnicity (%)	
NHW	76.6
NHB	12.1
Mexican Am	5.0
Other Hisp	1.9
Other/ Mixed Race	4.4
BMI (mean ± SD)	29.3 ± 5.1
Energy intake (kcal, (mean ± SD)	2114 ± 618
Daily fruit and vegetable intake, g p/d (mean ± SD)	599 ± 626
Pre-diabetes (%), *n* = 1193	
No	74.0
Yes	26.0
Diabetes (%)	
No	90.1
Yes	9.9
Hypertensive (%)	
No	64.8
Yes	35.2
Coronary heart disease or angina (pectoris) (%)	
No	95.0
Yes	5.0
Health insurance coverage (%)	
No	13.7
Yes	86.3
Family income to poverty ratio (%)	
Below 1	7.9
1–1.99	13.8
2–3.99	27.2
4 and above	51.2
Education level (%)	
HS/ GED and below	37.7
Some college and above	62.3
Smoking (%)	
Current smokers	28.6
Non-smokers with a history of smoking	26.2
Non-smokers	45.2

**Table 2:** Coronary heart disease prevalence by beverage intake frequencies among adults, ages 45–59 y – NHANES 2003–2006

		95%		Coronary Heart Disease^a^	
		Proportion	Confidence	% yes	*p*-value^3^
		%	Limits		
tEFF^b^ (ndSoda, FD, AJ) (*n* = 1230)					
≤ 3 times /mo	43.4%	40.3-	46.6%	3.3%	*0.044*
1–4 times /wk.	31.2%	28.1-	34.4%	5.1%	
5 times or more /wk.	25.4%	22.8-	28.3%	7.9%	
Non-diet soft drinks (*n* = 1230)					
≤ 3 times /mo	57.8%	54.9-	60.6%	4.1%	*0.037*
1–4 times /wk.	20.8%	18.5-	23.3%	3.9%	
5 times or more /wk.	21.4%	18.8-	24.3%	8.7%	
Diet soda (*n* = 1153)					
≤ 3 times /mo	56.3%	51.9-	60.5%	6.1%	0.406
1–4 times /wk.	15.3%	12.4-	18.7%	3.7%	
5 times or more /wk.	28.4%	24.4-	32.8%	4.3%	
Diet fruit drinks (*n* = 881)					
≤ 3 times /mo	85.7%	81.9-	88.9%	5.3%	0.286
1–4 times /wk.	07.7%	05.4-	10.9%	1.1%	
5 times or more /wk.	06.6%	04.7-	09.1%	5.5%	
Orange juice (*n* = 1230)					
≤ 3 times /mo	58.7%	54.2-	63.0%	5.9%	0.239
1–4 times /wk.	26.2%	22.7-	30.0%	3.2%	
5 times or more /wk.	15.1%	12.7-	18.0%	5.0%	

^a^Coronary Heart Disease (CHD) is defined as self-reported current or history of doctor diagnosed CHD and/ or angina pectoris [36]. ^b^tEFF refers to total excess free fructose beverages intake which includes any combination of high excess free fructose beverages for the NHANES period of 2003–2006 [35, 36]. This includes average daily intake of caffeinated and caffeine free non-diet soft drinks (ndSoda); non-diet fruit drinks (FD); and naturally high excess free fructose apple juice (AJ) [35, 36]. In 2003–2006 [the NHANES study period], high fructose corn syrup was the main sweetener in US soft drinks [31]. 3) Rao Scott Ҳ^2^ analysis was used to test for significance of differences in coronary heart disease prevalence by beverage intake frequency. A *p*-value <0.05 was considered significant and is indicated by italics

**Table 3:** Crude Associations between beverages intake frequencies and coronary heart disease in adults, Ages 45–59 y – NHANES 2003–2006

	Logistic Regression		
Coronary Heart Disease^a^	Crude OR	95% CI	*p*-value
tEFF^b^ (ndSoda, FD, AJ), n=1359			
apprx ≤3 times /mo	Reference -------		
apprx 1–4 times /wk	1.46	0.70–3.01	0.301
5 times or more /wk.	*2.61*	*1.11–6.11*	*0.029*
*n* = 1359			
Non-diet soft drinks, n=1359			
apprx ≤3 times /mo	Reference -------		
apprx 1–4 times /wk	0.88	0.40–1.94	0.747
5 times or more /wk.	*2.44*	*1.18–5.04*	*0.013*
*n* = 1359			
Diet soda, n=1273			
apprx ≤3 times /mo	Reference -------		
apprx 1–4 times /wk	0.55	0.22–1.36	0.188
5 times or more /wk.	0.70	0.35–1.40	0.300
*n* = 1273			
Diet fruit drinks, n=978			
apprx ≤3 times /mo	Reference -------		
apprx 1–4 times /wk	0.19	0.02–1.54	0.115
5 times or more /wk.	0.91	0.26–3.20	0.882
*n* = 978			
Orange juice, n=1359			
apprx ≤3 times /mo	Reference -------		
apprx 1–4 times /wk	0.46	0.20–1.03	0.060
5 times or more /wk.	0.79	0.41–1.53	0.474
*n* = 1359			

^a^Coronary heart disease (CHD) is defined as self-reported current or history of doctor diagnosed CHD and/ or angina pectoris [36]. ^b^tEFF refers to total excess free fructose beverages intake which includes any combination of high excess free fructose beverages for the NHANES period of 2003–2006 [35, 36], including average daily intake of caffeinated and caffeine-free non-diet soft drinks (ndSoda); non-diet fruit drinks (FD); and naturally high excess free fructose apple juice (AJ) [35, 36]. In 2003–2006 [the NHANES study period], high fructose corn syrup was the main sweetener in US soft drinks [31]. Crude OR's were considered statistically significant if the 95% confidence intervals did not include 1 and *p* values were <0.05, as indicated by italics

**Table 4:** Associations between beverages intake frequencies and coronary heart disease in adults, ages 45–59 y – NHANES 2003–2006

Coronary Heart Disease^c^	Multivariable Logistic Regression			Multivariable Logistic Regression		
	ratio	95% CI	*p*-value	ratio	95% CI	*p*-value
	^a^OR – adjusted for sex, race, age, BMI, SES, smoking, history of smoking, physical activity, total energy intake, total fruit and vegetable intake, hypertension, health insurance coverage			^b^OR – further adjusted for pre-diabetes, diabetes, glycated hemoglobin (A1C)		
tEFF^d^ (ndSoda, FD, AJ)	*n* = 1230			*n* = 1209		
apprx ≤3 times /mo	Reference -------			Reference -------		
apprx 1–4 times /wk	1.64	0.79–3.43	0.152	1.91	0.91–4.03	0.087
5 times or more /wk.	*2.34*	*1.01–5.40*	*0.047*	*2.82*	*1.16–6.84*	*0.023*
	^a^OR			^b^OR		
Non-diet soft drinks	n=1228			*n* = 1207	*n* = 1207	
apprx ≤3 times /mo	Reference -------			Reference -------		
apprx 1–4 times /wk	0.98	0.48–1.99	0.949	1.12	0.48–2.99	0.746
5 times or more /wk.	*2.01*	*1.01–4.00*	*0.048*	*2.18*	*1.12–4.23*	*0.023*
	^a^OR			^b^OR		
Diet soda	*n* = 1153			*n* = 1132		
apprx ≤3 times /mo	Reference -------			Reference -------		
apprx 1–4 times /wk	0.49	0.18–1.31	0.149	0.39	0.12–1.31	0.125
5 times or more /wk.	0.79	0.36–1.74	0.541	0.56	0.23–1.38	0.197
	^a^OR			^b^OR		
Diet fruit drinks	*n* = 881			*n* = 867		
apprx ≤3 times /mo	Reference -------			Reference -------		
apprx 1–4 times /wk	0.18	0.02–1.46	0.105	0.15	0.02–1.36	0.089
5 times or more /wk.	0.96	0.31–2.97	0.943	0.09	0.19–1.95	0.392
	^a^OR			^b^OR		
Orange juice	*n* = 1230			*n* = 1207		
apprx ≤3 times /mo	Reference -------			Reference -------		
apprx 1–4 times /wk	0.53	0.25–1.10	0.087	*0.45*	*0.26–0.77*	*0.005*
5 times or more /wk.	1.10	0.45–2.71	0.823	0.96	0.36–2.52	0.925

^a^Odds Ratio (OR) – adjusted for sex, race, age, BMI, SES, smoking, history of smoking, diabetes, glycated hemoglobin (A1C), physical activity, total energy intake, total fruit and vegetable intake, hypertension, health insurance coverage; ^b^Odds Ratio (OR) – further adjusted for pre-diabetes. ^c^Coronary heart disease (CHD) is defined as self-reported current or history of doctor diagnosed CHD and/or angina pectoris [36]. ^d^tEFF refers to total excess free fructose beverages intake which includes any combination of high excess free fructose beverages for the NHANES period of 2003–2006 [35, 36], including average daily intake of caffeinated and caffeine-free non-diet soft drinks (ndSoda); non-diet fruit drinks (FD); and naturally high excess free fructose apple juice (AJ) [35, 36]. In 2003–2006 [the NHANES study period], high fructose corn syrup was the main sweetener in US soft drinks [31]. Odds ratios were considered statistically significant if the 95% confidence intervals did not include 1 and *p *values were <0.05, as indicated by italics

Adults who consumed any combination of high fructose corn syrup sweetened soft drinks, fruit drinks and apple juice (tEFF) ≥ 5 times/wk. were more than two times (2.34) as likely to have CHD as 45–59 year olds who consumed tEFF ≤3 times/mo, independent of covariates (OR 2.34; 95% CI- 1.01–5.40; *P* = 0.047) (Table 4). Further adjustment for glycated hemoglobin, pre-diabetes, and diabetes, increased the odds among regular tEFF (≥5 times p/wk) consumers to nearly three times that of ≤3 times/mo consumers (OR 2.82; 95% CI - 1.16 – 6.84; *P* = 0.023) (Table 4). Regular (≥ 5 times/wk) consumption of HFCS sweetened soft drinks alone was similarly significantly associated with CHD (OR 2.18; 95% CI - 1.12–4.23; *P* = 0.023), independent of all covariates, including apple juice and fruit drinks. Moderate consumers (1–4 times/wk) of orange juice were half as likely to have CHD as seldom/never consumers (OR 0.45; 95% CI-0.26-0.77; *P* = 0.005), suggesting moderate consumption of orange juice may be protective against CHD (Table 4).

## Discussion

In this nationally representative sample, 45–59 year old adults who regularly consumed HFCS sweetened soft drinks had twice the likelihood of having CHD, independent of potential confounders, including diabetes and hypertension, while there was no association with diet drinks. The results of this cross sectional study are consistent with existing longitudinal epidemiology research [15, 16]. What differs in this study, relative to others, is that further adjustments were made for pre-diabetes and glycated hemoglobin (A1c). Notably, the association with CHD persisted after these adjustments, suggesting that the link between regular intake of HFCS sweetened soft drinks and CHD may be independent of blood glucose concentration.

Further, regular consumers of any combination of high excess free fructose beverages, including HFCS sweetened soft drinks, fruit drinks, and naturally high EFF apple juice were nearly three times more likely to have CHD than seldom or never consumers, independent of potential confounders, including pre-diabetes, glycated hemoglobin (A1c), and T2D status; higher than that for regular consumers of HFCS sweetened soft drinks alone. This is consistent with the hypothesis that regular intake of beverages with high ratios of fructose to glucose (excess free fructose) is associated with CHD. There was no association with low EFF beverages (diet drinks and citrus juice).

Although 5% or 10% more fructose in HFCS does not seem like a large amount [32, 33], HFCS that contains 60% or 65% fructose exceeds concentrations that are generally recognized as safe (55%) [34], and given average per capita intake (65 g/wk. or just under 1 lb./wk) [46, 47], it may be associated with above average fructose malabsorption (FM) in the general population [49–53]. Importantly, FM occurs after consumption of unpaired excess free fructose (EFF), but not with sucrose or equal monomers of fructose and glucose [49–51]. In the context of FM, these incremental amounts are significant. For example, in 65 g of HFCS (average per capita consumption) that is 55% fructose, there are 6.4 g of EFF. When the fructose content increases to 60%, the amount of EFF doubles to 13 g. When the fructose content increases to 65%, the amount of EFF triples to 19.4 g.

FM research indicates that 30% of healthy adults are FM positive after a 25 g EFF dose, and 10% are FM positive after a 12 g EFF dose, but not after consuming sucrose or equal amounts of fructose and glucose monomers [49]. Children are at increased risk of FM at lower EFF exposure [49–51]. Moreover, these amounts do not consider EFF contributions from naturally high EFF apple juice (67% fructose, 33% glucose) [35], apple juice based drinks [35], or foods and beverages sweetened with agave syrup, which contains more than 60% fructose [54]. Notably, one 8 oz. cup of apple juice contributes 9 g of EFF to the daily EFF load from HFCS [35], whereas the EFF contribution from orange juice is nominal (0.4 g).

From a glycemic perspective, orange juice, has a glycemic load (15) that is marginally lower than non-diet cola (16), and slightly higher than apple juice (12), per 250 ml serving [55]. Yet, analyses with orange juice were not associated with CHD, rather, moderate consumption appeared protective. Interestingly, research on SSB and T2D from the Black Womens’ Health Study – a prospective follow-up study of 59,000 African American women – showed that regular intake of orange and grapefruit juice was not associated with T2D – a common comorbidity of CHD [19]. Notably, other prospective studies with juice and T2D have not distinguished between juice types, and results have been mixed [12]. In a recent meta-analysis of epidemiologic research with T2D and sugary drinks (SSB, fruit drinks, and fruit juices), two studies that adjusted for glycemic index (GI) as a potential confounder showed that the increased risk of T2D persisted, independent of GI. None of the other studies in the meta-analysis adjusted for GI [12]. These results provide further evidence that another mechanism, independent of glycemic load and blood glucose, may be contributing to the increased risk of CHD, T2D and comorbidities among regular consumers of SSB.

From a total sugars perspective, orange juice (OJ) and HFCS sweetened cola are similar. Per 8 oz. cup, OJ contains 20.7 g of total sugars, and 10.1 g of total fructose [35], and cola contains 26.4 g of total sugars [35], and either 15.8 g (60% fructose), or 17.2 g (65% fructose) of total fructose, depending upon the HFCS formula used. However, per 8 oz. cup, cola contains 12 or 17.5 times the amount of EFF as OJ, depending upon the fructose percentage. Specifically, orange juice contains 0.4 g (NDB No. 09207) of EFF [35], whereas cola contains 4.7 g of EFF, when the HFCS formula contains 60% fructose, and 7.0 g of EFF when the HFCS formula contains 65% fructose; slightly lower than the 9 g in one cup of apple juice. Therefore, this substantial difference in EFF and underlying fructose malabsorption, rather than glycemic load, may explain why intakes of HFCS sweetened soft drinks, fruit drinks, and high EFF apple juice are associated with CHD, while intake of orange juice is not. More detail is available as Additional file 1: Table S2 and S3.

Notably, our prior epidemiologic research with high EFF beverages suggested that adults who regularly consumed HFCS sweetened soft drinks were nearly twice as likely to have chronic bronchitis as never/ seldom consumers [24], and that young adults who consumed any combination of high EFF beverages were three times as likely to have autoimmune arthritis as seldom/ never consumers, independent of lifestyle, dietary and socio-economic factors, diabetes and glucose status [25]. Our prior research with high EFF beverages and asthma/ chronic bronchitis was motivated by results of an HFCS food elimination diet [23], and research with young adult, auto-immune arthritis and CHD was motivated by the fact that chronic respiratory conditions are common comorbidities of arthritis and CHD [6–11].

Multiple hypotheses have been suggested to explain the mechanisms responsible for the association between SSB and chronic disease. In addition to glycemic load, researchers have hypothesized that high intake of fructose may increase the risk of CHD, because fructose increases triacylglycerol concentration, which promotes dyslipidemia – a risk factor for CHD. Researchers have also postulated that fructose consumption may promote endothelial dysfunction and vascular damage, possibly due to the production of uric acid, which may reduce endothelial nitric oxide and, thereby, increase oxidative stress [14–16]. However, our results suggest that these hypotheses do not fully explain the association between SSB and CHD. The higher probability of CHD among apple juice versus orange juice drinkers is not likely explained by antioxidant properties either, as post pasteurization vitamin C content is comparable between apple juice (95.5 mg/c) and orange juice (75 mg/c) [35]. We hypothesize that the mechanism involves malabsorption of unpaired excess fructose, as occurs when the fructose to glucose ratio exceeds 1:1.

Despite ongoing research, the exact cause of fructose malabsorption is not completely understood, but may result from EFF consumption that exceeds an individual’s EFF transport (GLUT5) capacity. [56] Notably, few natural foods contain significantly more fructose than glucose. Exceptions include apples, pears, watermelons, and mangoes [35]. According to the “fructositis” hypothesis, underlying fructose malabsorption and unabsorbed EFF may contribute to the intestinal in situ formation of AGE (enFruAGE). GI generated AGE may be an overlooked source of immunogens that travel to other tissues and promote inflammation by binding the pro-inflammatory receptor of advanced glycation end-products (RAGE) [23], known to be concentrated in the lungs, heart, connective tissues, lymph nodes [57] and, as recently discovered, the pancreas, in the presence of AGE [58, 59].

Intestinal enFruAGE may be an overlooked source of pro-inflammatory AGE that is separate from dietary AGE, and AGE that form in the systemic circulation of diabetes patients, under high blood glucose conditions [23]. The pH of the duodenum and jejunum, under high EFF conditions, appears to be more conducive to AGE formation than the systemic circulation of diabetes patients under high glucose conditions [23], and recent murine based research provides evidence of AGE formation in the jejunum [60, 61]. There is also evidence that the way excess free fructose is transported and absorbed into the body differs than when fructose is consumed in relatively equal proportions with glucose [62]. For many people, consumption of excess free fructose results in unabsorbed fructose in the intestines. Importantly, the intestinal environment after a meal may be highly conducive to AGE formation in the presence of unabsorbed excess free fructose, as transition metals including iron (Fe2+) are known to accelerate the Maillard reaction [63, 64]. Further, anionic ligands including the phosphates in soft drinks, particularly colas, and the bicarbonate from pancreatic juice, are potent catalysts of glycation at specific sites on proteins [65–68].

There is evidence that AGE are deposited in arterial walls, accumulate over time, and contribute to stenosis and atherosclerosis [20]; AGE accumulate in the coronary artery of heart disease patients with [20] and without [69, 70] diabetes and contribute to CHD [20, 69, 70]; AGE accumulate in the pancreas and contribute to pancreatitis and impaired ß-cell function; [58, 59] AGE are elevated in connective tissues including the synovium, sub-lining, and cartilage of autoimmune arthritis sufferers [71–75]; and the receptor of advanced glycation end-products (RAGE) is a key mediator of asthma [76].

The interaction of AGE with the receptor for advanced glycation end products (RAGE) is well studied. Cytokines, known to be associated with AGE/RAGE pro-inflammatory signaling, are involved in the inflammatory process in asthma and other comorbid conditions, including RA, CHD and T2D [9]. There is evidence that regular consumption of SSB elevates the same pro-inflammatory cytokines associated with AGE/ RAGE pro-inflammatory signaling. For example, in the Health Professionals Follow-up study with 42,883 men, intake of HFCS sweetened, but not artificially sweetened beverages, was significantly associated with higher C-reactive protein (CRP), IL6, and tumor necrosis factor receptors [15]. These biomarkers are consistent with the transcriptional activation of genes associated with AGE/ RAGE/ pro-inflammatory signaling and are known to be involved with chronic inflammation [75]. Importantly, a similar investigation of sucrose found no association with CRP, and high consumption of sucrose-sweetened foods and drinks had only a limited association with CRP [77].

Notably, our study of EFF in children showed that regular consumers of any combination of high EFF beverages were five times more likely to have asthma as seldom/ never consumers; and that moderate (1–4 times/wk) and regular (≥5 times/wk) apple juice consumers were more than twice as likely to have asthma as seldom/never consumers. There was no association with orange juice [26]. It is possible that enFruAGE contribute to inflammation beginning in childhood, as inflammation is commonly observed in children with asthma [9], including mild and moderate asthma [78]. EnFruAGE may begin to accumulate in arterial walls and in the pancreas in childhood, as recent research indicates that acute pancreatitis is more common in children than previously thought [70, 79]. The intestinal in situ formation of pro-inflammatory enFruAGE, resulting from regular EFF consumption, could explain why children with asthma have an increased risk of T2D, and why adults with asthma have an increased risk of T2D and CHD. It is a potential pathway that could explain the connection between the gut and asthma, idiopathic chronic bronchitis, RA, T2D, and CHD.

Importantly, while this study was undergoing peer review, results of a proof of concept experiment were published, which provide evidence of possible enteral enFruAGE formation. Incubation of amino acids with fructose - but not glucose - at concentrations and pH that would be present in the intestines after a meal, led to a time and dose dependent formation of AGE intermediates, as measured by fluorescence, after just 1 h of incubation – a time frame well compatible with the digestive process [80].

This study is subject to limitations. First, the associations are cross-sectional, limiting causal inference. Although cross sectional and longitudinal studies are both observational studies, cross sectional studies provide only a single “snapshot” in time. Second, the NHANES data have some degree of error which may have affected our results. Third, our models did not include adjustment for triglycerides or LDL - known risk factors of CHD - as these were measured only in a subset of respondents. Fourth, fruit and vegetable intake - a covariate included in analyses - may be underestimated, as responses that measure intake of individual foods do not always capture intake from mixed dishes. However, our results are consistent with existing largescale study results, wherein intake of HFCS sweetened soft drinks, and fruit drinks increased CHD risk, independent of dietary quality [15, 16]. Fifth, CHD status in NHANES is based on self-report, so there is potential for reporting bias. However, our results are comparable to findings from existing large-scale longitudinal studies of HFCS sweetened soft drinks and CHD. [15, 16] Sixth, high EFF beverages are only one food category that contributes to daily EFF load. Associations between dietary EFF and CHD may be higher when other food sources of EFF are considered. For example, a 20 oz. (590 ml) bottle of cola contains 65 g (240 kcal) from HFCS. Coincidentally, average daily HFCS intake during the study period was approximately 65 g/d. However, only adolescent boys consumed this amount of HFCS (kcal/d) from SSB (12–19 y boys, 273 kcal; girls, 138 kcal), relative to other age groups: (2–5 y boys, 71 kcal; girls, 70 kcal; 6–11 y boys, 141 kcal; girls 112 kcal; 20–39 y men, 252 kcal; women, 138 kcal; 40–59 y men, 159 kcal; women, 86 kcal; 60 y ≥ men, 70 kcal; women 42 kcal) [43]. Therefore, for many people, HFCS sweetened foods (other than beverages) may be major sources of high fructose corn syrup. Lastly, the extent to which high EFF agave syrup and crystalline fructose – increasingly popular HFCS alternatives – are contributing to fructose malabsorption prevalence is unknown.

## Conclusion

The results presented here support the proposed hypothesis. HFCS sweetened soft drinks, fruit drinks, and apple juice may contribute to CHD, a common comorbidity of chronic respiratory conditions and autoimmune arthritis, possibly due to the high ratio of fructose to glucose in these beverages. Underlying fructose malabsorption, may contribute to the intestinal in situ formation of pro-inflammatory advanced glycation end-products (enFruAGEs), which are eventually absorbed and induce inflammation of the coronary arteries [23]. Longitudinal studies, clinical and biochemical research is needed to clarify and confirm the mechanisms involved.

## Additional file

Additional file 1: Table S1. 2003–2006 NHANES questions regarding physical activity and metabolic equivalent (MET) scores provided for each activity. **Table S2.** Characteristics of US average daily intakes of high fructose corn syrup (HFCS). **Table S3.** Characteristics of high fructose corn syrup (HFCS) sweetened soft drinks, 100% fruit juices, and diet drinks. (DOCX 19 kb)

## Abbreviations

A1c: Glycated hemoglobin
ADI: Average Daily Intake
AGE: Advanced Glycation End-products
BMI: Body Mass Index
CHD: Coronary Heart Disease
COPD: Chronic Obstructive Pulmonary Disease
CRP: C Reactive Protein
EFF: Excess Free Fructose
enFruAGE: Extracellular newly identified fructose associated Advanced Glycation End-products
FFQ: Food Frequency Questionnaire
FM: Fructose Malabsorption
GI: Gastro-Intestinal tract
GRAS: Generally Recognized as Safe
HFCS: High Fructose Corn Syrup
MET: Metabolic Equivalent
NHANES: National Health and Nutrition Examination Survey
RA: Rheumatoid Arthritis
RAGE: Receptor of Advanced Glycation End-products
SES: Socio-Economic Status
SSB: Sugar sweetened beverages
T2D: Type 2 Diabetes
tEFF: Any combination of high fructose corn syrup sweetened soft drinks, fruit drinks, and apple juice
