# Supplementary material for: Intake of high fructose corn syrup sweetened soft drinks, fruit drinks and apple juice is associated with prevalent coronary heart disease, in U.S. adults, ages 45–59 y
Source: BMC Nutr. 2017 Jun 27;3:51. doi: 10.1186/s40795-017-0168-9 (PMC7050890; doi:10.1186/s40795-017-0168-9)
Supplement: Supplementary file 1 — 2003–2006 NHANES questions regarding physical activity and metabolic equivalent (MET) scores provided for each activity. Table S2. Characteristics of US average daily intakes of high fructose corn syrup (HFCS). Table S3. Characteristics of high fructose corn syrup (HFCS) sweetened soft drinks, 100% fruit juices, and diet drinks. (DOCX 19 kb) [file 40795_2017_168_MOESM1_ESM.docx]

**Table S1. 2003 – 2006 NHANES questions regarding physical activity and metabolic equivalent (MET) scores provided for each activity.**

| **Physical activity questions and corresponding MET scores.^1^** | **Met Score^2^** |
| --- | --- |
| You sit during the day and do not walk about very much. | 1.4 |
| You stand or walk about a lot during the day, but do not have to carry or lift things very often. | 1.5 |
| You lift light loads or climb stairs or hills often. | 1.6 |
| You do heavy work or carry heavy loads. | 1.8 |
| Done moderate activities that caused light sweating or moderate increases in breathing or heart rate including brisk walking, bicycling for pleasure, golf, and dancing, etc. | 3.5 |
| Have you over the past 30 days, walked or bicycled to get to/from work | 4.0 |
| Done tasks in or around home or yard that required moderate or greater physical effort | 4.5 |
| Done vigorous activities that caused heavy sweating or large increases in breathing or heart rate including exercise, sports, and other active hobbies. | 7.0 |

^1^Physical activity was obtained by asking, “Which of four sentences best describes your usual daily activities?”^[36]^ ^2^For analysis purposes, Metabolic Equivalent (MET) scores were summed and divided into three equal quantiles.

**Table S2. Characteristics of US average daily intakes of high fructose corn syrup (HFCS)**

| **Source** | **Total Sugars** | **Total Fructose** | **Total Glucose** | **Fructose to Glucose Ratio** | **^4^Excess Free Fructose** | **^5^Glycemic Load**  **p/ 250 ml** |
| --- | --- | --- | --- | --- | --- | --- |
| **^1^ADI of HFCS (55% fructose/ 45% glucose)** | 65 g | 35.7 g | 29.3 g | 1.2: 1 | 6.4 g | 16 |
| **^1^ADI of HFCS (60% fructose^2^/ 40% glucose)** | 65 g | 39 g | 26 g | 1.5 : 1 | 13 g | <16 |
| **^1^ADI of HFCS (65% fructose^3^/ 35% glucose)** | 65 g | 42.2 g | 22.8 g | 1.9 : 1 | 19.4 g | <16 |

1)The source of average daily intake (ADI) of High Fructose Corn Syrup is the US Department of Agriculture.^[46-47]^ Existing research indicates that the fructose used to sweeten HFCS sweetened soft drinks is higher than the 55% that is generally recognized as safe (GRAS). 2-3)Two studies found the fructose content in HFCS used to sweeten non-diet soft drinks was 60% ^[32]^ and 65% ^[33]^. 4)Excess free fructose is defined as fructose in excess of glucose, as occurs when the fructose to glucose ratio exceeds 1:1. 5)The source of glycemic load is the International Tables of Glycemic Index and Glycemic Load Values: 2008. The glycemic load likely decreases, as the fructose to glucose ratio increases. ^[54]^

**Table S3. Characteristics of high fructose corn syrup (HFCS) sweetened soft drinks, 100% fruit juices, and diet drinks**

| **Source** | **^1^NDB entry No.** | **^1^Total Sugars p/ 8 ounce cup in grams** | **^1^Total Sucrose p/ 8 ounce cup in grams** | **^1^Total Fructose p/ 8 ounce cup in grams** | **^1^Total Glucose p/ 8 ounce cup in grams** | **Fructose/ Glucose ratio** | **^2^Excess Free Fructose p/ 8 ounce cup in grams** | **^3^Glycemic Load**  **p/ 250 ml** |
| --- | --- | --- | --- | --- | --- | --- | --- | --- |
| **HFCS sweetened cola (55% fructose/ 45% glucose)** | 14148 | 26.4 | 0.0 | 14.5 | 11.9 | 1.2: 1 | 2.6 | 16 |
| **^5^HFCS sweetened cola (60% fructose/ 40% glucose)** | **^5^**unavailable | 26.4 | 0.0 | 15.8 | 10.6 | 1.5: 1 | 5.2 | ^4^16 |
| **^5^HFCS sweetened cola (65% fructose/ 35% glucose)** | **^5^**unavailable | 26.4 | 0.0 | 17.2 | 9.2 | 1.9: 1 | 8.0 | ^4^16 |
| **Any combination of HFCS sweetened soft drinks and fruit drinks; and apple juice** | - | varies | varies | varies | varies | varies | varies | varies |
| **Apple juice** | 09400 | 24.0 | 3.1 | 15.7  (14.2g + 1.5g from sucrose) | 8  (6.5g + 1.5g from sucrose) | 2:1 | 7.4 | 12 |
| **Grapefruit raw^6^** | 09112 |  |  |  |  | 1:1 | 0.0 |  |
| **Orange juice** | 09207 | 20.7 | 10.1 | 11.0  (6.0g + 5.0g from sucrose) | 10.6  (5.6g + 5.0g from sucrose) | 1:1 | 0.4 | 15 |
| **Pineapple juice** | 09409 | 24.9 | 3.8 | 11.4  (9.5g + 1.9g from sucrose) | 13.5  (11.6g + 1.9g from sucrose) | .8:1 | 0.0 | 15 |
| **Grape juice** | 09130 | 35.9 | 0.1 | 18.6 | 17.2 | 1:1 | 1.4 | unavailable |
| **Diet soft drinks and diet fruit drinks** | - | 0.0 | 0.0 | 0.0 | 0.0 | - | 0.0 | 0 |

1)Total sugars are derived from the US National Nutrient Database for Standard Reference – a freely accessible reference database.^[35]^ 2)Excess free fructose (EFF) is defined as unpaired fructose that is in excess of glucose (total fructose minus total glucose); fructose malabsorption occurs after consumption of EFF or fructose only, but not after consumption of sucrose.^[48-52][55]^ 3)Glycemic load is estimated by multiplying the food’s listed glycemic index (GI) value with glucose as the reference food by the listed g carbohydrate per serving and dividing by 100.^[54]^ 4) The glycemic load of HFCS sweetened cola that contains 60% or 65% fructose may be different than the glycemic load with 55% fructose, as glycemic load likely decreases, as the fructose to glucose ratio increases. 5) Existing research provides evidence that the fructose in SSB is higher than generally recognized as safe (GRAS). Independent labs found 60% fructose and 65% fructose in popular soft drinks, as measured during two different studies – not the 55% that is GRAS.^[32-33]^ 6)Total sugars including sucrose, fructose and glucose were available for raw grapefruit, not grapefruit juice. Therefore, the fructose to glucose ratio is provided for grapefruit juice, but not sugars breakdown.^[35]^
